# Supplementary material for: Allele-specific Expression Reveals Multiple Paths to Highland Adaptation in Maize
Source: Mol Biol Evol. 2022 Nov 3;39(11):msac239. doi: 10.1093/molbev/msac239 (PMC9692238; doi:10.1093/molbev/msac239)
Supplement: msac239_Supplementary_Data [file msac239_supplementary_data.zip › 06_Upload_any_supplementary_information_files/Supplementary_Figures.docx]

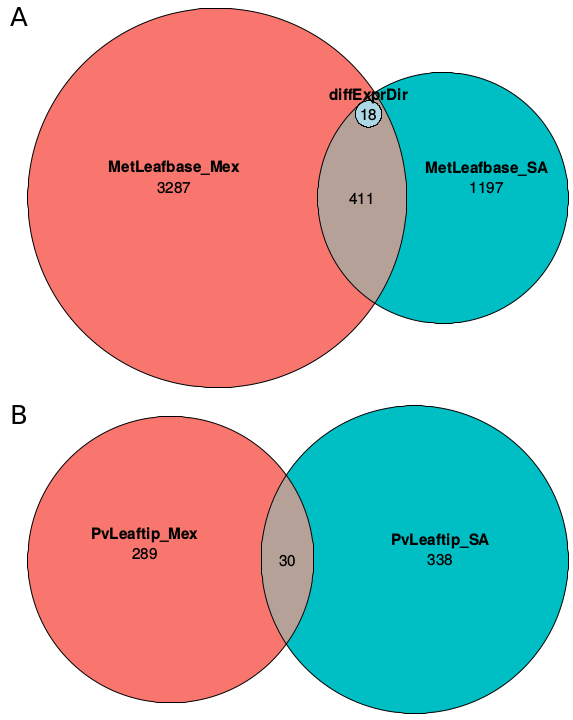


**Supplementary Figure 1.** Numbers of differentially expressed genes between highland and lowland populations from Mexico (red) and South America (blue) and common genes detected in both continents. Analyses of F1 samples from the two populations were done separately post-normalization. (A) MetLeafbase and (B) PvLeaftip. The small inset in the overlapping region of figure A, shows genes significant in both populations, but with opposite directions of expression change.


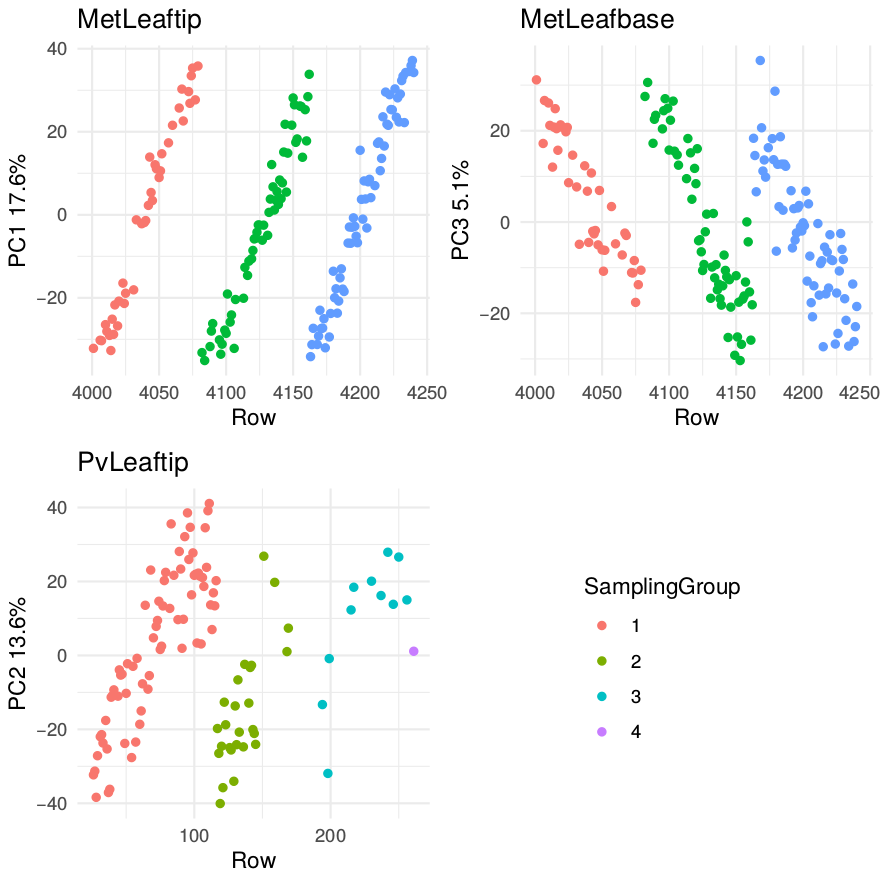


**Supplementary Figure 2.** Associations between principal components of gene expression and sampling times within the 1.5 hr sampling window for the three site:tissues. Each dot represents one F_1_ sample. Points are arranged on the x-axis according to the field row (rows snaked back and forth across the field in a set of 5 (Metepec) or 10 (Puerta Vallarta) ranges). Colors represent the 3-4 sampling teams that sampled plants in parallel during the sampling window. For this figure we chose the PC with the strongest association with Row (i.e., order of sampling) to demonstrate that this was an important component of expression variation.

~~
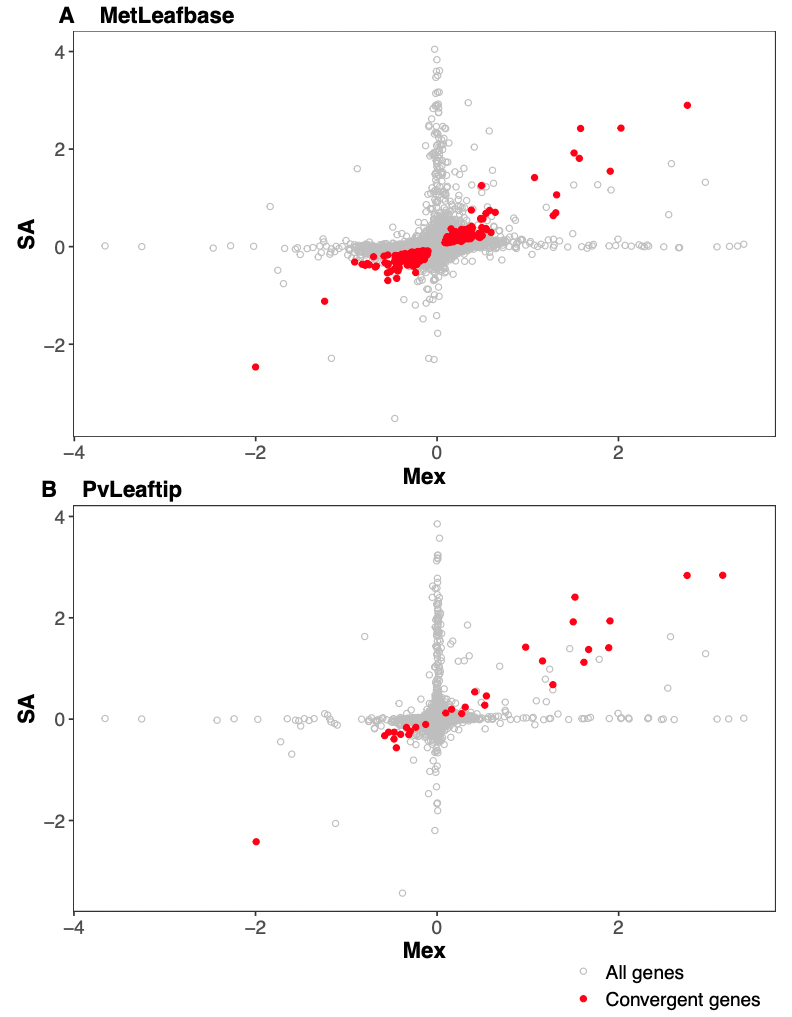
~~

**Supplementary Figure 3.** Correlation of posterior mean highland effects between Mexican and South American population for all genes measured for gene expression (in gray) and a subset of genes showing evidence of convergent evolution (in red) in (A) MetLeafbase and (B) PvLeaftip.

**
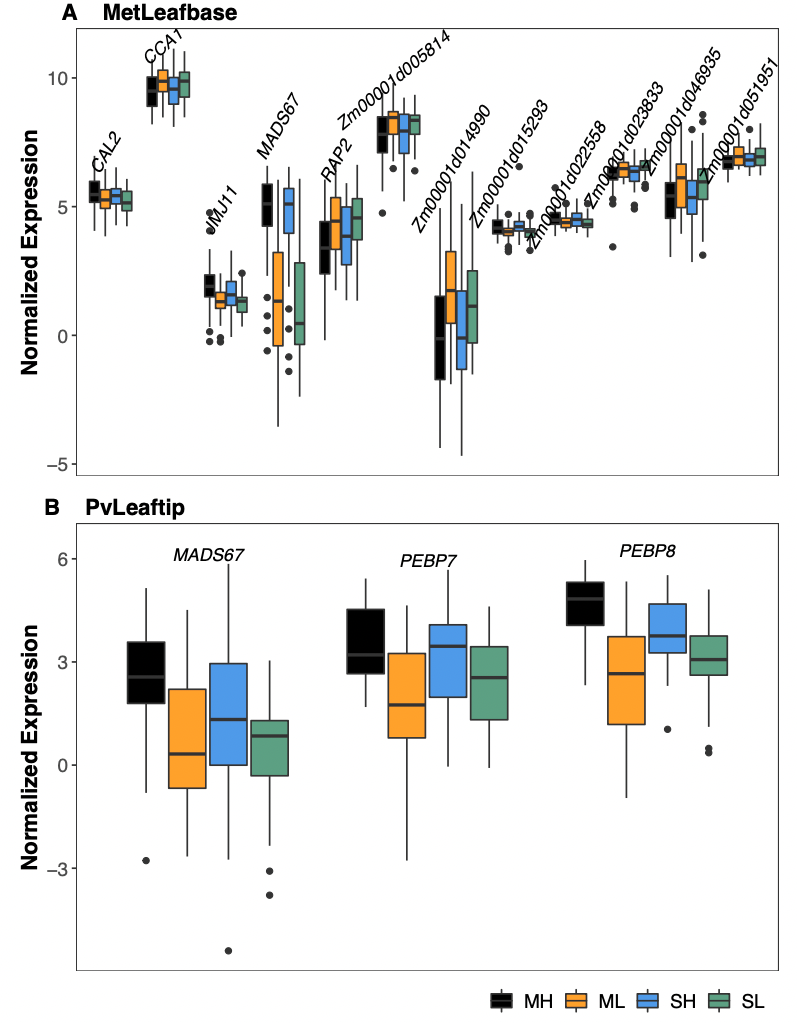
**

**Supplementary Figure 4** Expression of flowering-related genes in the Mexican Highland (ML), Mexican Lowland (ML), South American Highland (SA), and South American Lowland (SL) populations in A) MetLeafbase and (B) PvLeaftip. These flowering-related genes are identified by looking for overlapping between the convergent genes and maize flowering time candidate genes aggregated by Li et al. (2016) and Swarts et al. (2016).

**
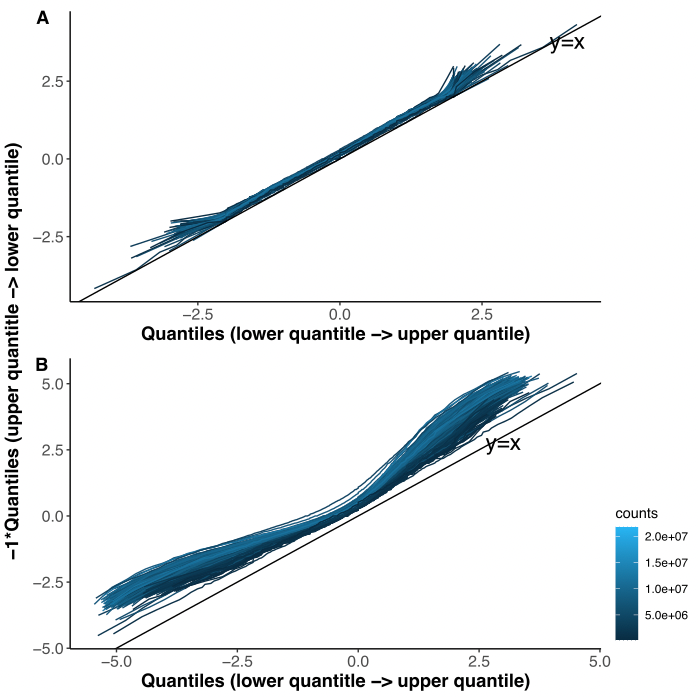
**

**Supplementary Figure 5.** Filtering for SNPs with at least one count from both alleles reduces reference bias in ASE ratios. Q-Q plots of quantiles of log2ASE ratios across all genes (x axis) against -1 * the quantiles of log2ASE ratios in reverse order (y axis). Reference bias (tendency of higher counts for the reference allele) shows up as up-ward shifts relative to the y=x line. (A) filtered: heterozygous SNPs for each sample, and (B) unfiltered: all heterozygous SNPs for each sample where both alleles were detected, the total number of reads overlapping the SNP was at least 10, and the absolute value of the log2ASE ratio (i.e, log2(Landrace/B73)) was no larger than 2.


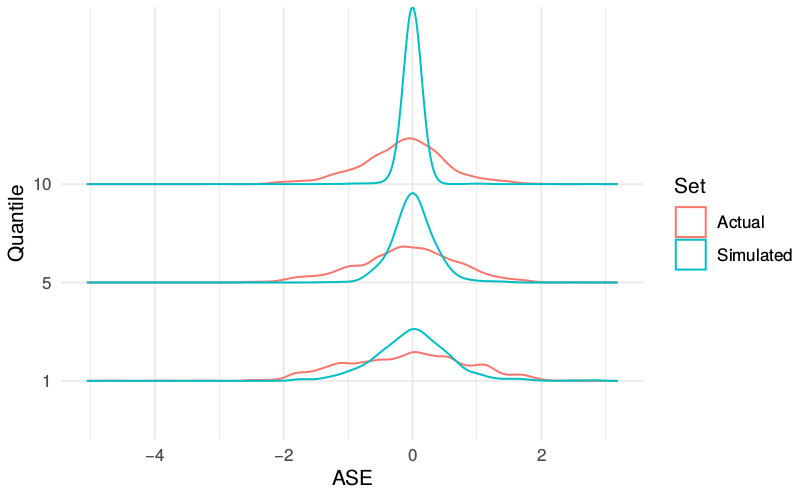
**Supplementary Figure 6.** Distributions of ASE from real data (real ASE, in blue) and from simulated data (in red) for a random sample. We first stratified the real ASE by the average expression level of the genes (quantile 1= lowest expressed, 10=highest); and then, for each gene, we simulated ASE by sampling from a binomial distribution given its total count.


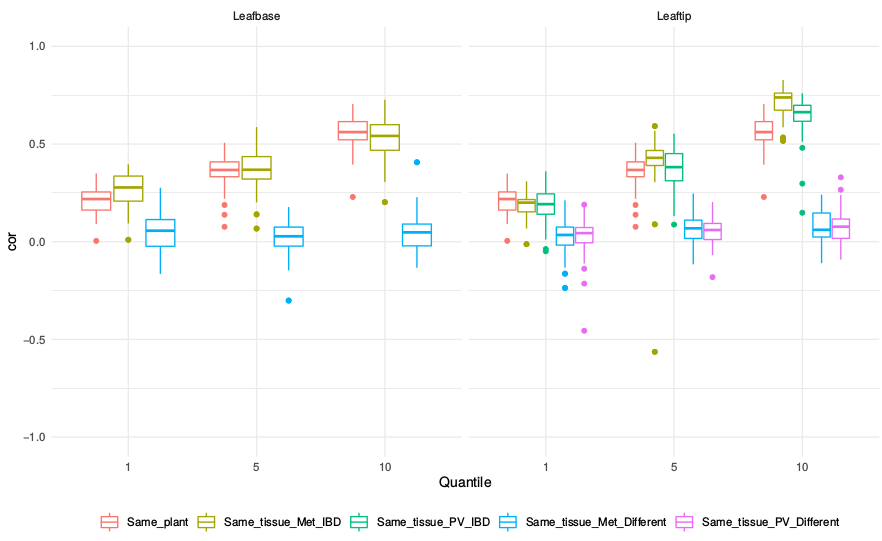
**Supplementary Figure 7.** Correlation of ASE estimates across samples. Genes are stratified by deciles of expression level (1=lowest, 10=highest), and correlations are reported comparing ASE between different tissues collected from same plant (red), between two individuals of the same F_1_ family from the same tissue for genes in genomic regions where the two individuals shared the same haplotype (IBD) (yellow = both plants in Metepec, green = one plant in Metepec, one in Puerta Vallarta) or did not share the same haplotype (blue/purple).


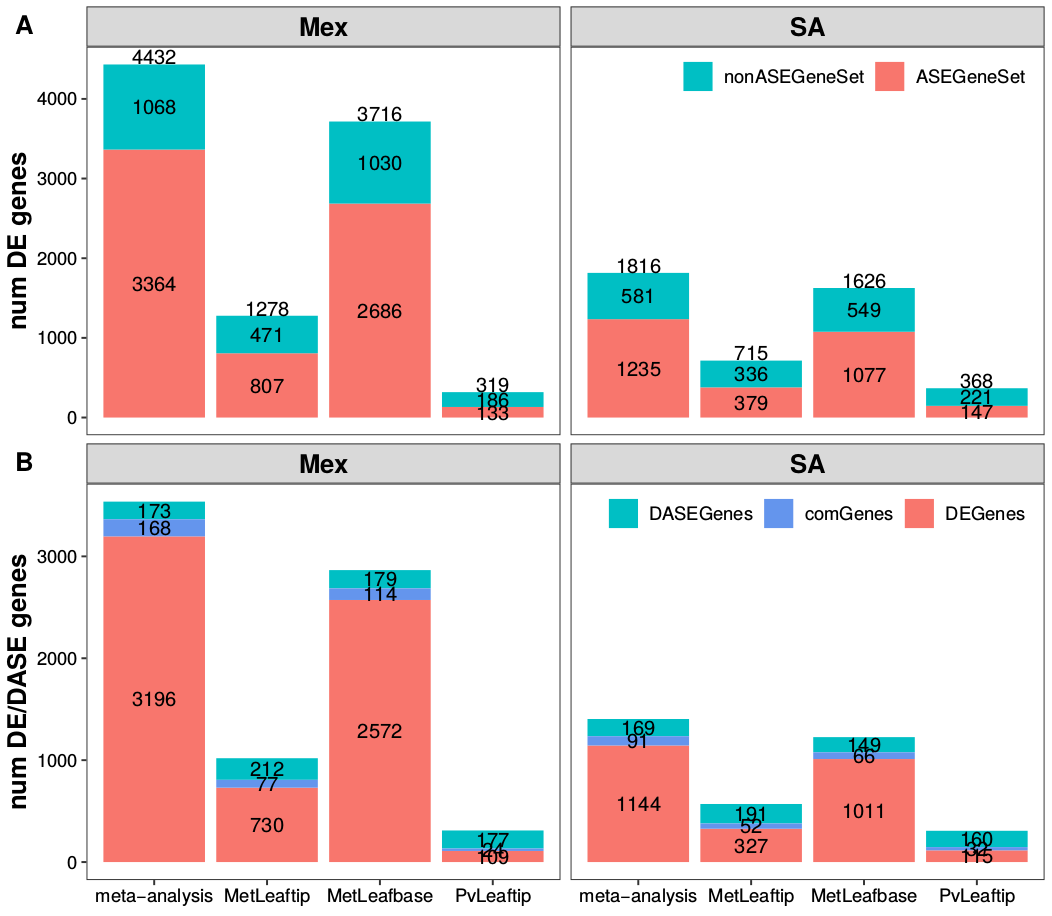


**Supplementary Figure 8.** Comparison of the number of genes detected in the differential gene expression (DGE) and differential allele specific expression (DASE) analyses. (A) Numbers of differentially expressed (DE) genes detected in single-tissue analysis and in a meta-analysis based on the same set of genes for DASE analysis (colored in red) and additional genes that were assayed for gene expression but not for ASE (colored in blue). (B) Comparison of DE genes and DASE genes detected in single tissue analysis and in a meta-analysis based on the same set of assayed genes. The numbers of common genes detected between DASE and DE analyses were highlighted in light blue.


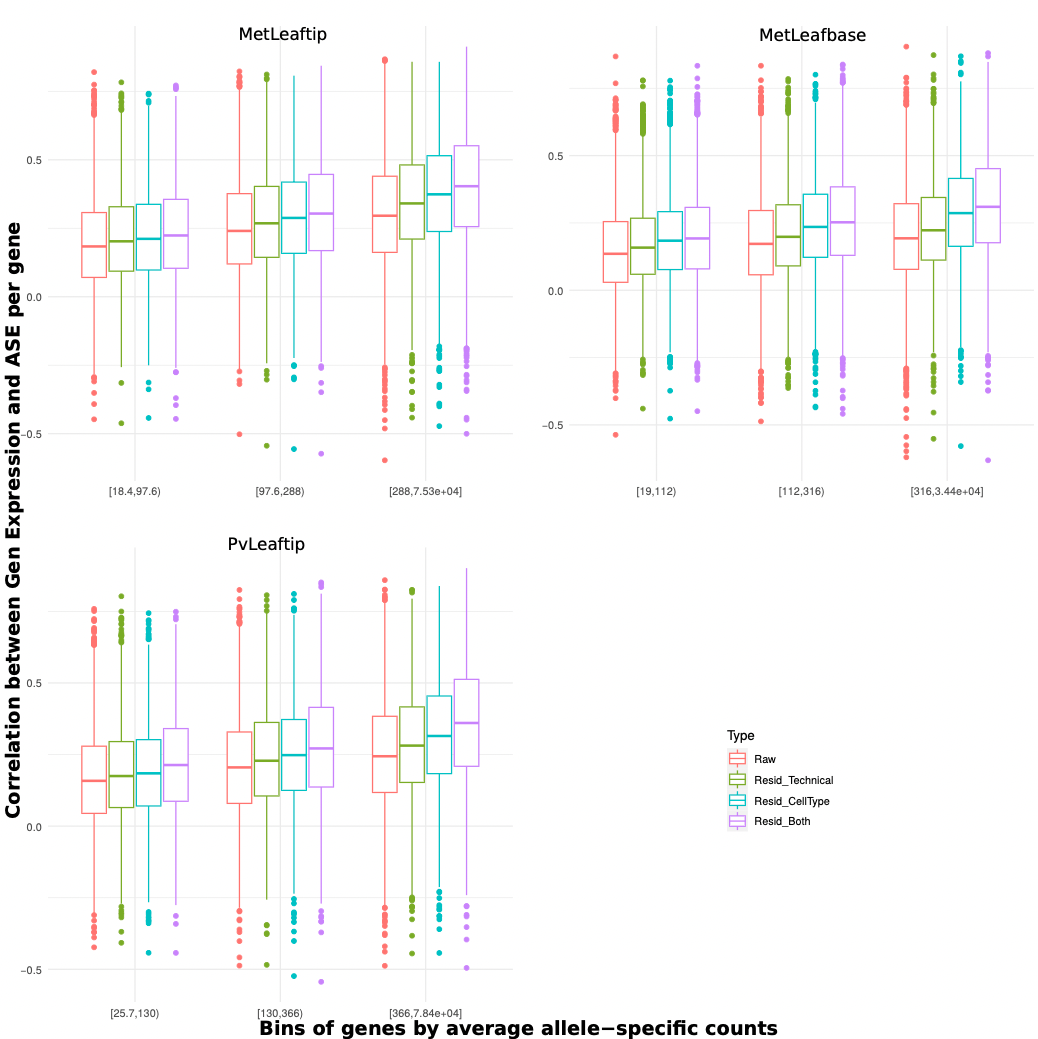


**Supplementary Figure 9.** Correlations between log2 scale ASE and log2 scale gene expression for common genes assayed for gene expression and ASE in each site:tissue. Boxplots show the distributions of correlations across genes. Genes were stratified by terciles of expression level.
